# Editorial: Functional Metagenomics for Enzyme Discovery

**DOI:** 10.3389/fmicb.2022.956106

**Published:** 2022-06-23

**Authors:** Vanessa A. Varaljay, Trevor C. Charles, Rolf Daniel

**Affiliations:** ^1^Air Force Research Laboratory, Wright-Patterson Air Force Base, OH, United States; ^2^Department of Biology, University of Waterloo, Waterloo, ON, Canada; ^3^Department of Genomic and Applied Microbiology, Institute of Microbiology and Genetics, Georg-August-University of Göttingen, Göttingen, Germany

**Keywords:** functional metagenomics, enzymes, protein expression, activity, uncultured microorganisms, biotechnology, bioprospecting

This Research Topic highlights the diverse approaches used to discover microbial and viral enzyme functions by leveraging metagenomics particularly from uncultured microorganisms. These state-of-the-art approaches illustrate how functional metagenomics uncovers novel enzymes and activities relevant to environmental ecology and climate, bioremediation, host health and disease, and importantly to biotechnology based on samples collected and analyzed from the gut, rumen, compost, and acid mine drainage systems among others.

The first article of this Topic Wang et al. used functional metagenomics screening of a fosmid library to identify active glycoside transporters in uncultured bacteria in the human gut. This research sheds light on the transport mechanisms that microorganisms use to select for specific natural oligosaccharides. Glycosides play critical roles in health and disease but few transporters have been experimentally validated. Interestingly, the authors used growth as a screening method due to the very low secretion potential of *E. coli* (Kleiner-Grote et al., 2018) and with this approach they were able to identify 10 active clones with transporters. The authors concluded the hit rate for active clones is low, but this result is in line with the findings of other studies also discussed by Lu et al. The study brought to light the need for automation screening facilities for higher throughput and this along with suggestions to explore cell-free methods by Keown et al. could be the future of functional metagenomics studies for enzyme discovery.

In Barrett et al., carbohydrate metabolism was analyzed through carbohydrate active enzymes (CAZymes) which play a critical role in cattle rumen and even climate change. Through changes in feed diet, notably an extreme diet high in concentrate and without grass or corn, the methane emissions from the Holstein cows were significantly reduced. Based on sequence-driven approaches also used in part by Lu et al., Keown et al., and Vidal et al., the authors concluded that changes in the microbiome and specifically CAZymes (xylan-related) likely played a role in this decrease of methane production. This analytic approach using CAZy subgroupings is a novel way to look at functional and taxonomic analysis in the rumen metagenome with important implications for climate change.

Both Lu et al. and Vidal et al. used and emphasized the importance of complementary function-driven and sequence-based approaches for their studies on bioprospecting novel lipolytic enzymes and esterases. In Lu et al., the authors focused on lipolytic enzymes which have industrial and biotechnological applications. In the sequence-based approach, the authors used and developed specific Hidden Markov models to mine for lipolytic enzymes in over 175 metagenomes and 15 habitats from online databases. They found that there were differences shaped by habitat and found that lipolytic enzymes are constrained by ecology and within distinctive microbial groups. In the function-driven approach, Lu et al. used *E. coli* plasmid libraries and uncovered 115 unique lipolytic enzymes, 7 of which could not be grouped in any known family, in compost samples which they determined were a reservoir of lipolytic enzymes. Significantly this study is the first to consider lipase ecology, function, and phylogeny collectively with respect to novel lipase identification using multiple functional metagenomics strategies.

In Vidal et al., the authors used a slightly different approach by focusing on esterases in Acid Mine Drainage systems (AMDs) which are acidic environments. Here, they stressed that enzymes in AMDs have been greatly uncharacterized and likely have significant value for biotechnology applications. In the sequence-based approach, they used homology searches with BLAST methods and in the function-driven approach, they screened *E. coli* fosmid libraries; both approaches resulted in 16 esterases and a subset of these was also experimentally tested. Structural analyses using the *de novo* AlphaFold2 tool (Jumper et al., 2021) provided potential explanations of the enzyme biochemistry including thermostability. Structural predictions and analyses will continue to refine structure-function relationships critical in functional metagenomics studies as was also mentioned in Keown et al.

Keown et al. took a different focus by mining for DNA polymerase (PolA) from viruses and used a sequence-based approach to find diversity from metagenomics studies. DNA polymerases are important in biotechnology applications such as sequencing. The authors acknowledged that obtaining function for viral genes is challenging but that functional metagenomics fills the knowledge gap. They mined many online databases specifically for viral genomes and metagenomes and selected 48 PolA sequences for synthesis. Interestingly they were able to expand the diversity of functional PolA sequences and concluded that selection pressure due to lifestyle (for e.g., lytic, lysogenic) drives the biochemical diversity of viral DNA polymerases.

Functional metagenomics for enzyme discovery, especially from extreme environments and uncultured microorganisms, has made great strides since Handelsman et al. (1998) and the articles published as part of this Research Topic highlight these advancements. There are challenges with optimizing screening and expression platforms which were illustrated in all of the studies in this Research Topic. However, the studies in this Research Topic underscore the importance of how function-driven and sequence-based functional metagenomics are able to access a sequence diversity beyond what can be obtained with cultivation studies alone. With continued improvements in sequencing, structural analyses, and functional screening and protein expression, this field will only continue to expand the diversity of enzyme function with far reaching implications for medicine, biotechnology, and climate change.

## Author Contributions

VV drafted the article. TC and RD provided critical revisions and approved final version to be published. All authors contributed to the article and approved the submitted version.

## Author Disclaimer

The views and opinions presented herein are those of the author(s) and do not necessarily represent the views of Department of Defense (DoD) or its Components. Appearance of, or reference to, any commercial products or services does not constitute DoD endorsement of those products or services. The appearance of external hyperlinks does not constitute DoD endorsement of the linked websites, or the information, products or services therein.

## Conflict of Interest

TC is founder and CEO/CSO of Metagenom Bio Life Science Inc. The remaining authors declare that the research was conducted in the absence of any commercial or financial relationships that could be construed as a potential conflict of interest.

## Publisher's Note

All claims expressed in this article are solely those of the authors and do not necessarily represent those of their affiliated organizations, or those of the publisher, the editors and the reviewers. Any product that may be evaluated in this article, or claim that may be made by its manufacturer, is not guaranteed or endorsed by the publisher.
